# Balancing of Resonant Differential Coils for Broadband Inductive Sensor Systems

**DOI:** 10.3390/s24186009

**Published:** 2024-09-17

**Authors:** Liam A. Marsh, Adam D. Fletcher, Anthony J. Peyton

**Affiliations:** Department of Electronic and Electrical Engineering, University of Manchester, Manchester M13 9PL, UK; adam.fletcher@manchester.ac.uk (A.D.F.); a.peyton@manchester.ac.uk (A.J.P.)

**Keywords:** inductive sensor systems, resonant coils, gradiometer, differential coil pair, metal detection, NDT, magnetic induction spectroscopy, broadband sensor systems

## Abstract

Differential coils are frequently implemented in inductive sensing systems. They can be considered as a single coil that is made up of two or more subcoils, wound in series opposition. They can be used on the transmit or receive side of measurement systems, and, if designed correctly, ensure no coupling between coils under background conditions. By cancelling background coupling, the receive electronics only needs to be able to measure the change in coupling produced by a target. This allows for a more efficient use of the dynamic range, and for larger receive-side amplifier gain, thereby improving SNR. When subcoils are not electrically similar, it can be hard to engineer the coil to be perfectly balanced across a wide bandwidth. This paper presents an analytical model of a resonant differential coil pair that is tested and applied on a planar metal detector for the detection of buried objects. The model demonstrates the capability to balance an arbitrary differential coil pair, which has a broad applicability across a range of inductive sensor applications such as metal detection and non-destructive testing. The method is applied to the practical system. The results show that the correction resulting from this method ensures a stable balance across a significantly enhanced bandwidth. In the case studied here, the bandwidth of the experimental system is increased from 20 kHz to 90 kHz.

## 1. Introduction

**Figure 1:**
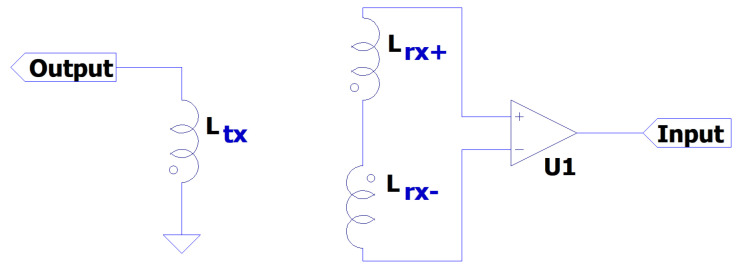
Schematic of the gradiometer showing the relative phases of each inductive element.

Many inductive measurement systems rely on detecting the change in coupling between two or more coils. Such systems are used for a variety of applications such as walk-through security screening, detection of buried or visibly obscured metallic objects, detecting contaminants in the food and drug industry, and in surveying [1,2,3,4]. Such configurations are also used for eddy current non-destructive testing [5]. These systems generally feature a “transmit” coil, which is excited with an AC current of one or more frequencies. This current generates a magnetic field in the surrounding area. Operating frequencies are typically dictated by the application, with conventional “metal detection” systems operating in the range of 100 Hz–1 MHz [6,7,8]. This magnetic field induces a voltage in any nearby “receive” coils. The magnitude and phase of the induced signal is a superposition of direct coupling and any secondary fields caused by eddy currents circulating in targets [9]. The primary field is needed to induce these eddy currents. The magnitude of the induced current, and thus the secondary field, is proportional to the magnitude of the primary field. Therefore, to maximise SNR, it is often desired to have the largest transmit current that the system can support, subject to other constraints (e.g., operation on batteries or safe limits of EM exposure [10]). However, only changes in this field contain useful information about targets within the sensing region. Consequently, it becomes necessary to design coil geometries that reduce the background coupling between transmit and receive coils, i.e., in the absence of a measurable target. “Gradiometer” coils are used in such applications. These consist of networks of two or more “subcoils” that are connected in series. An example of this is shown in Figure 1, where $L_{tx}$ denotes a transmit coil, and $L_{rx+}$ and $L_{rx-}$ denote two halves of a pair of receiver coils; although in practice, the transmit–receive roles can be reversed. The figure shows the general form of an arbitrary gradiometer coil arrangement, which also describes a high-level view of the system used in this study. In this figure, a “balanced” system would be defined where the voltage induced in coil $L_{rx+}$ is equal in magnitude and opposite in phase to $L_{rx-}$. Constructing coils in this way reserves almost all of the dynamic range of the receive network for identifying changes in the coupling, rather than having to reserve large portions for the directly coupled signal.

In many sensing applications, it is common for the secondary signals produced by targets to be small, e.g., sub 10 mV [11]. The detection of such signals typically requires receive circuit gains of at least 100 to allow such signals to be measurable over any background noise, and to make the most efficient use of the noise profile of instrumentation amplifiers [12]. Taking this value as an example, and assuming an instrumentation amplifier running off a ±5 V supply, the background signal coupled from the transmit to the receive coil cannot exceed 50 mV, and ideally should be much less than this to remain within the dynamic range of the amplifier. One option might be to reduce the level of the primary signal. For example, by reducing the current in the transmit coil. However, this would lower the sensitivity of the system, as the response of targets is proportional to the magnitude of the applied primary field. Therefore, it is crucial for coil arrays to be designed to minimise background coupling between the transmit and receive coils.

Gradiometer coils are used in a large range of sensing applications, and consist of a range of geometries [1,13,14,15]. Measurement systems sensing objects in free space (e.g., walk-through metal detection) can often be nulled by means of constructing gradiometer coils with identical pairs of coils wound in anti-phase.

As the two coil halves have very similar electrical parameters, they generally produce good cancellation of the primary signal across their full inductive ranges. However, different coil geometries are typically needed for other sensing applications that require some measurement of the sensing environment. For example, measuring soil in which metallic components are buried or measuring the response of large conducting bodies. In such cases, the coil network must be nulled with respect to the primary signal, while also being capable of measuring the properties of the environment around the sensor. Therefore, they cannot be nulled to the far-field effects. Such systems typically have asymmetric coil geometries, e.g., concentric coils with different turns ratios, which are widely used in sub-surface detection systems [13,16,17]. As the subcoils have different sizes and numbers of turns, their electrical parameters can be substantially different from one another. Although it is still straightforward to design a coil that is nulled at a specific frequency in such circumstances, this occurs when the in-phase and quadrature components of each side of the differential coil pair fully cancel. Achieving this balance across a wide range of frequencies is generally unachievable due to the fact that parasitics represent complex, frequency-dependent impedances that are difficult to optimise. Therefore, many inductive detection systems are only nulled at a specific frequency and inherently imbalanced across a broader range of frequencies. By achieving broadband balancing of differential coil pairs, it is possible to design spectroscopic measurement systems that can be used for more advanced applications such as object classification.

**Figure 2:**
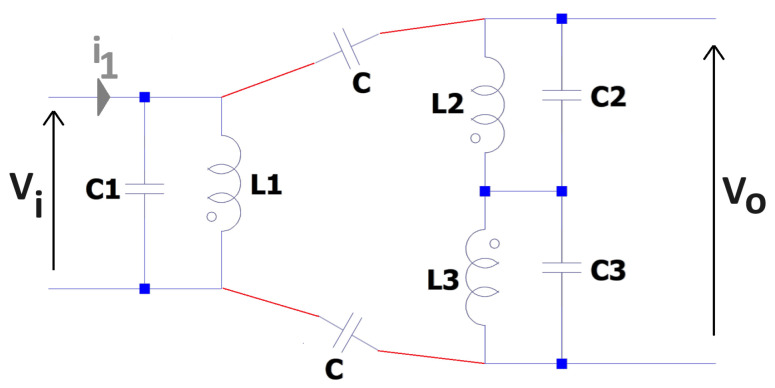
Conceptual view of parasitic capacitance.

An ideal inductor is often considered to be a passive component that consists of complex impedance with no real part, e.g., 0+jXΩ. However, in practice, inductors have other intrinsic and parasitic impedance characteristics that alter their behaviour when considered experimentally. A “real” inductor has a number of associated considerations, which typically include a series resistance that is associated with the electrical properties of turns within the inductor. As a wire-wound inductor has a defined length, cross-sectional area, and resistivity, there will be an associated series resistance, often termed R_s_, i.e., R+jXΩ, where R≠0. There is further parasitic behaviour as there are a number of routes for parasitic capacitative coupling; this includes action between coils and between turns within a coil [18,19,20]. Figure 2 shows an example of the methods of visualising the parasitic capacitance terms that may exist in a coil network, as in Figure 1, as lumped equivalent components.

This paper considers a simplified model of a lumped circuit model that is described in Section 3. This considers the associated R, L, and C terms that can be used to describe a first-order approximation to a practical inductor. In doing so, the “stray” capacitance that exits between coil pairs (labelled ‘C’ on Figure 2) is neglected. This is because such values are very weak when compared with both the inductive coupling and the equivalent parallel capacitance (labelled C1, C2, and C3 in Figure 2), which models the turns of the inductors. The reason for this is that the parallel capacitances C1–3 originate due to bundles of closely wound turns of wire with a thin enamel coating; this has a much greater capacitative effect than stray coupling between coils that are separated by up to several centimetres. The results presented in this paper will show that this is an appropriate assumption when considering the bandwidth of the system considered in this paper, which is shown to be approximately 0.1 kHz ≤f≤ 100 kHz.

## 2. Description of Sensor System

**Figure 3:**
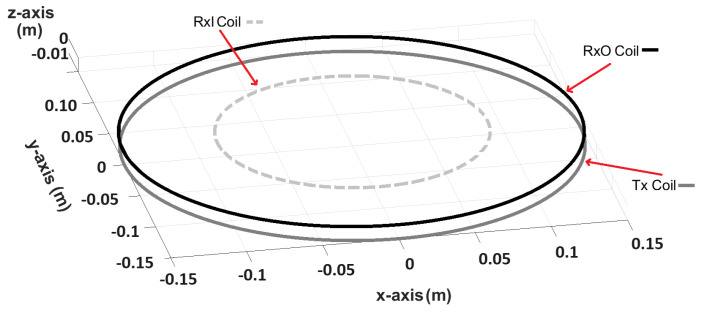
Coil geometry of the inductive sensing system.

**Figure 4:**
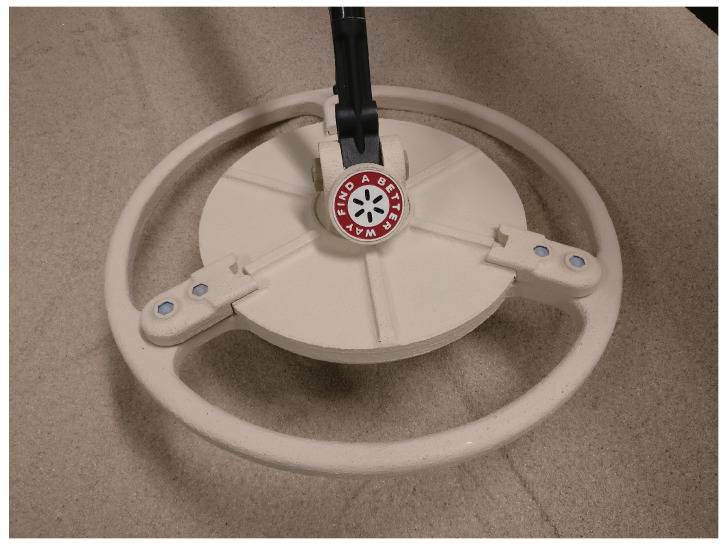
Image of the assembled sensor head.

The coil geometry considered in this paper was previously reported in [21]. It is a concentric arrangement consisting of a circular transmit coil (Tx) with a diameter of 0.3 m, an “outer” receive coil (Rx_O_) with a diameter of 0.3 m, and an “inner” receive coil (Rx_I_) with a diameter of 0.089 m. The turns ratio is 4.23, with the inner receive coil having a greater number of turns. The inner receive coil is wound in anti-phase with respect to the outer receive coil and the transmit coil. The array is shown in Figure 3 and an image of the sensor system is shown in Figure 4. The coils were wound around acrylic formers and “balanced” while being driven by a multi-frequency transmit signal with components from 3.8 kHz to 27.87 kHz. The coils were then encapsulated inside a mould using epoxy resin; this ensures that the coils remain mechanically stable and protected from damage. However, an unavoidable consequence of this process is that the balance is disrupted by the buoyancy generated by the liquid resin during the initial pour, and then by the contraction of the resin as it cures. The resulting receive coil pair becomes slightly mis-balanced as a result. The coils can no longer be driven with the desired multi-frequency signal without saturating the receive stage. Consequently, further correction is required.

## 3. Equivalent Circuit Models

**Figure 5:**
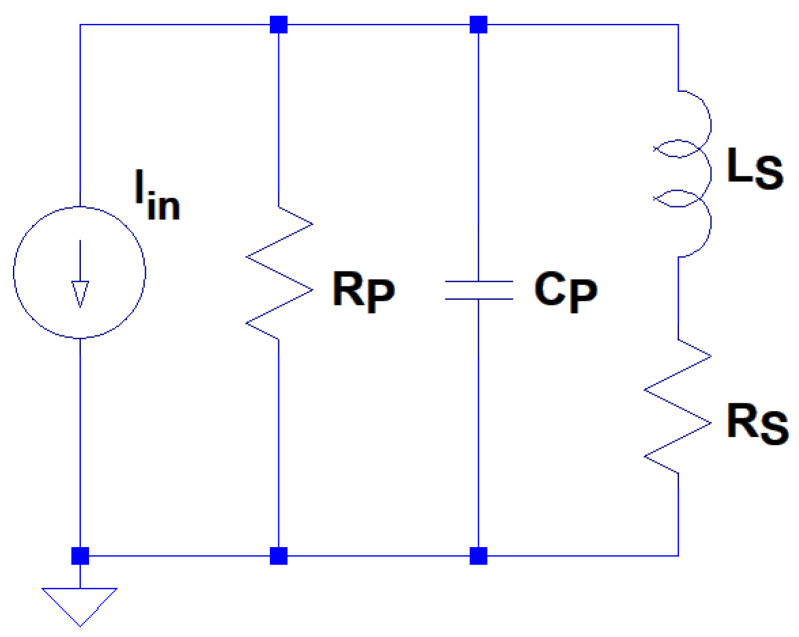
Equivalent circuit model for a resonant inductor.

A resonant coil can be modelled with a lumped equivalent RLC circuit, as shown in Figure 5. In practice, the terms for R_P_, L_S_ and C_P_ are distributed over the turns of each coil. Consequently, the lumped circuit approach is a first order approximation of a more complicated electromagnetic problem. Nevertheless, as this paper will show, the lumped equivalent model is sufficient to provide both an understanding of the dominant effects and an ability to optimise the system.

The R, L, and C terms correspond with certain characteristics of the impedance spectrum of a coil, typically when considered on a log–log scale. The DC resistance of the coil, R_s_ is dominant at low frequencies. As $|j\omega L|\gg |R_{s}|$ the inductive response tends to dominate, which is represented by a linear increase, corresponding to jωL. At very high frequencies, $|{\left(j\omega C\right)}^{-1}|\gg |j\omega L|$, and so the coil behaves in a capacitative manner, with impedance decreasing as a function of frequency. Resonance occurs in the region where $|{\left(j\omega C\right)}^{-1}|\approx |j\omega L|$. This occurs when the inductive terms and capacitative terms, which are opposed in phase by $180^{\circ }$, fully cancel in magnitude to eliminate the complex term in the impedance, i.e., R+j0Ω. A pole appears in the impedance spectrum, which corresponds to this resonant behaviour. Consequently, it can be very unpredictable to operate an inductive system at or near resonance, as there are very sharp gradients involved. By definition, the same is also true of operating near zeroes, which are also shown to occur in the system considered in this paper. The Q-factor of the resonant peak is defined as the ratio of the peak height to its width. This dictates the gradient and associated bandwidth of the resonant peak, and so also needs to be considered when analysing the behaviour of a coil. The phase of the impedance also varies as the resistive, inductive, and capacitative terms dominate.

An ideal inductive sensing system would consist only of inductive coupling. However, for a practical system, each of these terms must be considered in order to describe the behaviour of a system. Therefore, it is necessary to determine the appropriate parameters of the model via experimental means.

**Figure 6:**
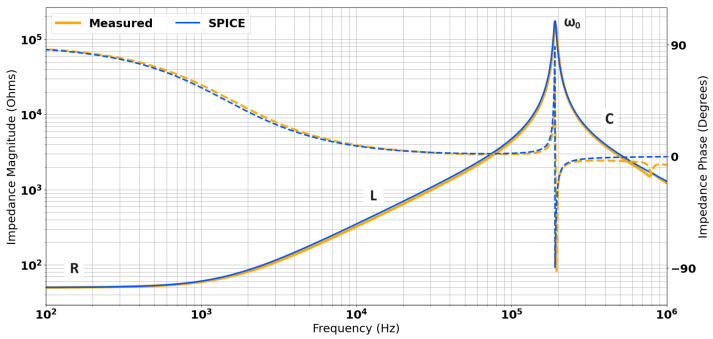
Comparison of measured and simulated impedances using the equivalent circuit model for the inner receive coil.

The self impedance of each discrete coil was measured using a Solartron 1260 impedance analyser [22]. The response of the inner receive coil (Rx_I_) is shown in Figure 6. The values for series resistance (R_s_) can be taken from the low frequency impedance, labelled as ‘R’ in Figure 6. The value for $L_{s}$ is calculated using the gradient of the “inductive” region of the spectrum (indicated by L in Figure 6). The parasitic capacitance $C_{P}$ can be calculated from the resonant frequency ($\omega_{0}$) and the known value for $L_{s}$ using the following:

(1) $$\begin{array}{c} \omega_{0}=\frac{1}{2\pi \sqrt{L_{s}C_{P}}} \end{array}$$

which results from an analysis of Figure 6 when the damping resistance is ignored. Finally, the damping resistance $R_{P}$ can be estimated by matching the impedance magnitude at the resonant peak. These lumped parameter values for each of the three coils were extracted from Solartron measurements of each coil individually. These were then used in an LTSpice (see [23]) simulation. Figure 6 shows that for the inner receive coil, the measured data and the equivalent circuit show excellent agreement across the frequency range.

## 4. Analytical Model for a Resonant Gradiometer

**Figure 7:**
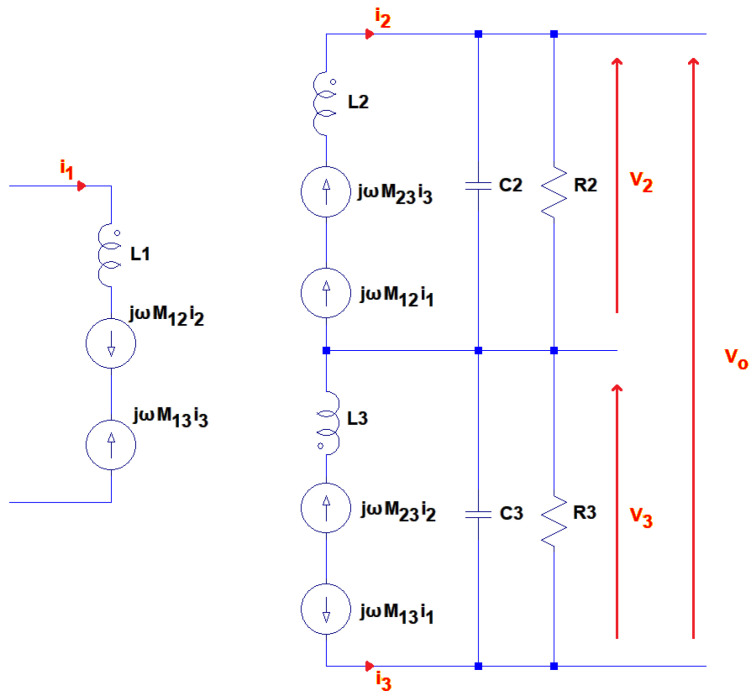
Schematic for the analytical model.

The equivalent circuit for the coil network has been modelled analytically using circuit theory. Figure 7 shows the equivalent circuit model and includes mutual inductance terms, $M_{xy}$, to represent the coupling between the coils in the network. In the figure, L1 represents the transmit coil, and L2 and L3 represent the inner and outer receive subcoils, respectively. The coupling is modelled through the addition of current sources that relate the current in each coil with each mutual inductance.

The geometry of the gradiometer considered in this example means that the inner and outer coils are only weakly coupled. Therefore, $M_{13}\gg M_{23}$, so receive side terms proportional to this can be neglected. Furthermore, given that the transmit coil is excited, then $i_{1}\gg i_{2}$ or $i_{3}$, the transmit terms proportional to these factors are also ignored.

The voltage across the L2 arm of the receive coil network, $V_{2}$, can be calculated by evaluating the impedance of the network as in (3).

(2) $$\begin{array}{cc} V_{2} & =\frac{\frac{R_{2}\frac{1}{j\omega C_{2}}}{R_{2}+\frac{1}{j\omega C_{2}}}}{\frac{R_{2}\frac{1}{j\omega C_{2}}}{R_{2}+\frac{1}{j\omega C_{2}}}+j\omega L_{2}}j\omega M_{12}i_{1} \end{array}$$

(3) $$\begin{array}{cc} \; & =\frac{j\omega M_{12}i_{1}}{1+\left(j\omega \right)\frac{L_{2}}{R_{2}}+{\left(j\omega \right)}^{2}L_{2}C_{2}} \end{array}$$

Similarly, for $V_{3}$:

(4) $$\begin{array}{c} V_{3}=\frac{j\omega M_{13}i_{1}}{1+\left(j\omega \right)\frac{L_{3}}{R_{3}}+{\left(j\omega \right)}^{2}L_{3}C_{3}} \end{array}$$

Therefore, the voltage across the gradiometer $V_{o}$ can be expressed using (5) and (6).

(5) $$\begin{array}{cc} V_{o} & =V_{2}-V_{3} \end{array}$$

(6) $$\begin{array}{ccccccc} \; & \; & \; & \; & \; & \; & =j\omega \frac{M_{12}g_{3}\left(j\omega \right)-M_{13}g_{2}\left(j\omega \right)}{g_{2}\left(j\omega \right)g_{3}\left(j\omega \right)}i_{1} \end{array}$$

where

(7) $$\begin{array}{c} g_{i}\left(j\omega \right)=1+\left(j\omega \right)\frac{L_{i}}{R_{i}}+{\left(j\omega \right)}^{2}L_{i}C_{i} \end{array}$$

This can be converted to a transimpedance by dividing by the current in the transmit coil, $i_{1}$ in (8).

(8) $$\begin{array}{cc} \frac{V_{o}}{i_{1}}= & j\omega \frac{\left(M_{12}-M_{13}\right)+\left(j\omega \right)\theta +{\left(j\omega \right)}^{2}\phi }{g_{2}\left(j\omega \right)g_{3}\left(j\omega \right)} \end{array}$$

where

(9) $$\begin{array}{cc} \theta & =M_{12}\frac{L_{3}}{R_{3}}-M_{13}\frac{L_{2}}{R_{2}} \end{array}$$

(10) $$\begin{array}{cc} \phi & =M_{12}L_{3}C_{3}-M_{13}L_{2}C_{2} \end{array}$$

As the coils are close to balance, it is possible to aggregate the mis-balance about a common coupling and a term, ΔM, which quantifies the mis-balance. So, assuming $M=M_{12}=M_{13}$ and $\Delta M\;=\;M_{12}-M_{13}$, Equation (8) can be expressed as (11):

(11) $$\begin{array}{cc} \frac{V_{o}}{i_{1}}= & j\omega \Delta M\frac{\left[1+\left(j\omega \right)\left(\frac{M}{\Delta M}\frac{L_{3}}{R_{3}}-\frac{M}{\Delta M}\frac{L_{2}}{R_{2}}\right)+{\left(j\omega \right)}^{2}\left(\frac{M}{\Delta M}L_{3}C_{3}-\frac{M}{\Delta M}L_{2}C_{2}\right)\right]}{g_{2}\left(j\omega \right)g_{3}\left(j\omega \right)} \end{array}$$

(12) $$\begin{array}{cc} = & j\omega \Delta M\frac{1+j\omega X+{\left(j\omega \right)}^{2}Y}{g_{2}\left(j\omega \right)g_{3}\left(j\omega \right)} \end{array}$$

This substitution means that the numerator term linear in jω can be written as (13) and the term quadratic in jω becomes (14):

(13) $$\begin{array}{c} X=\frac{1}{\Delta M}\left(M\left(\frac{L_{3}}{R_{3}}-\frac{L_{2}}{R_{2}}\right)+\frac{\Delta M}{2}\left(\frac{L_{3}}{R_{3}}+\frac{L_{2}}{R_{2}}\right)\right) \end{array}$$

(14) $$\begin{array}{c} Y=\frac{1}{\Delta M}\left(M\left(L_{3}C_{3}-L_{2}C_{2}\right)+\frac{\Delta M}{2}\left(L_{3}C_{3}+L_{2}C_{2}\right)\right) \end{array}$$

**Figure 8:**
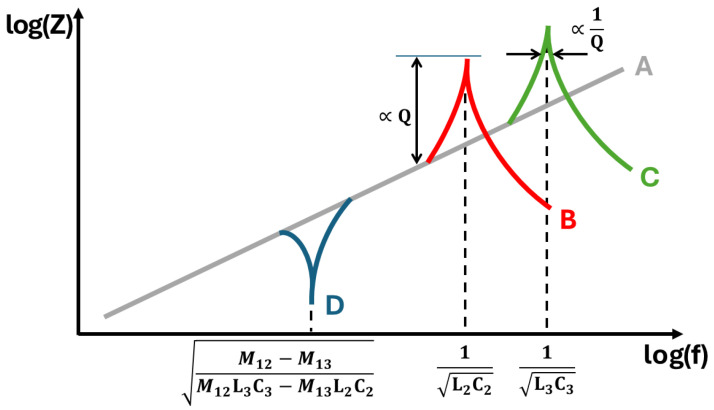
Features of the impedance spectrum of a resonant gradiometer.

The analytical model contains a single zero and two poles, shown schematically in Figure 8. The colours used to represent the features in the figure are the same as the terms in (8), which produce corresponding characteristics of A–D, respectively. The underlying linear trend, “A” is the “Faraday Ramp”, jω; this represents an ideal inductor. The poles occur at the resonant frequencies of each receive subcoil, specified as “B” and “C”. These correspond to the two $g_{i}$ functions in the denominator of (8). As the denominator is a product of these functions, then setting either of these terms equal to zero will yield a pole. The transimpedance zero occurs at point “D”, when the numerator of (8) is equal to zero.

The zero is problematic for broadband balancing of coils for a number of reasons. Firstly, the non-linearity impacts the stability when operating within this region and limits the inductive region of the impedance spectrum as defined in Figure 6. This fundamentally limits the bandwidth of the coil array. Secondly, it has a phase transition, which results in a $180^{\circ }$ phase shift between frequencies either side of the zero. This means that it is not possible to reduce magnetic coupling by introducing a variable transmit–receive transformer on a split ferrite core. In such circumstances, there is no complete optimisation; reducing coupling below the zero inherently improves coupling above the zero, and vice versa.

The coil geometry is dictated by the sensor system’s application, e.g., the physical size of the sensor, the expected targets to be detected, and the separation between the sensor and target. Consequently, coil geometry cannot be changed without compromising the design of the system in some way. This means that the values of *L* are fixed and parasitic capacitance cannot be changed. For this reason, it is not possible to increase bandwidth by pushing the poles to higher frequencies. The bandwidth will always be limited by the lowest resonant frequency of the subcoils in the gradiometer, as indicated by “B” in Figure 8. However, the frequency at which these poles occur can be decreased through the addition of parallel capacitance, or damped by adding parallel resistance. This process is normally exploited in single frequency inductive systems to take advantage of the improved SNR that occurs at resonance [24]. However, this approach is not applicable for multi-frequency systems, as they cannot be tuned to single frequency operation.

Unlike the poles, the zero term can be moved both higher and lower in frequency. This is because it is dependent on the resonant balance, Q-factor balance, and magnetic balance in the numerator of (13) and (14). Under conditions of ideal balancing, each of the terms in the numerator of (8) would be identical for both coils:

$$\mathrm{Magnetic}\;\mathrm{balance}:M_{12}=M_{13}$$

$$\mathrm{Q-factor}\;\mathrm{balance}:\frac{L_{2}}{R_{2}}=\frac{L_{3}}{R_{3}}$$

$$\mathrm{Resonant}\;\mathrm{balance}:L_{2}C_{2}=L_{3}C_{3}$$

In the case of Q-factor balance, the terms in (8), which are linear in jω (also shown in (13)) simplify to $\frac{L_{3}}{R_{3}}$ and the terms of second order in jω in (shown in (14)) simplify to $L_{3}C_{3}$,

Therefore, the numerator simplifies to (15) that cancels out the second pole as the term is repeated in the denominator of (11)

(15) $$\begin{array}{c} \left(1+j\omega \frac{L_{3}}{R_{3}}+{\left(j\omega \right)}^{2}L_{3}C_{3}\right) \end{array}$$

Under these conditions of balance, (11) simplifies to become

(16) $$\begin{array}{c} \frac{V_{o}}{i_{1}}=j\omega \frac{\Delta M}{\left(1+j\omega \frac{L_{2}}{R_{2}}+{\left(j\omega \right)}^{2}L_{2}C_{2}\right)} \end{array}$$

## 5. Simulation of the Coil Array

**Figure 9:**
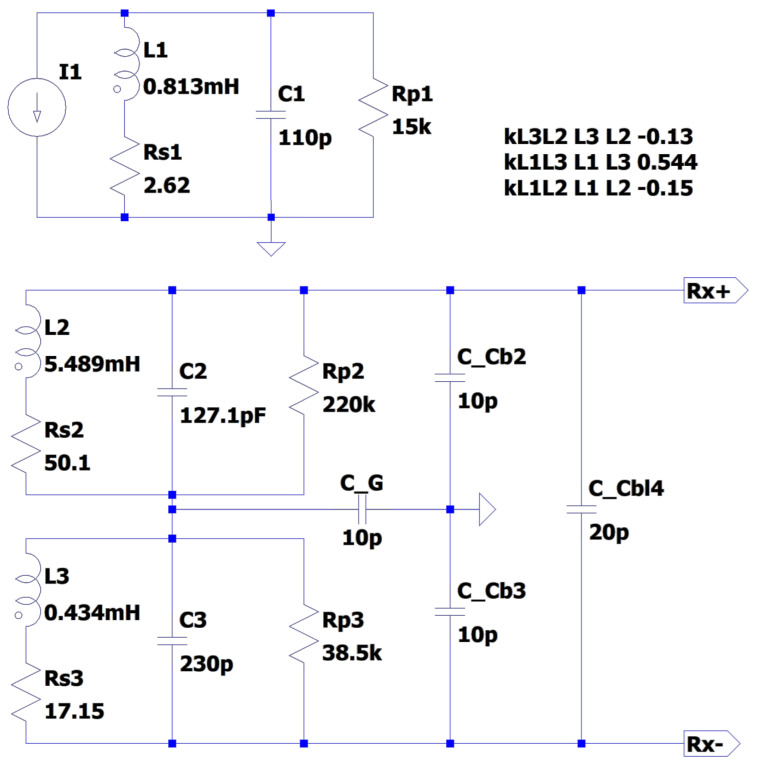
SPICE model of the equivalent transimpedance of the coil network before correction.

In order to assess the performance of the analytical model, an LTSpice model of the equivalent circuit was developed. This is illustrated in Figure 9. The lumped component parameter values for each coil were extracted from experimentally measured spectra (as in Figure 6) and a representative capacitance (C_Cb2 etc) for the connecting cable was taken from the manufacturer’s specification.

All component values in the equivalent circuit for both the analytical model and the SPICE model were kept constant at the values that were measured or calculated from empirical data. However, the coil coupling coefficients could not be measured in the same way. In order to estimate these, an iterative method was used to minimise the error between the measured data and the simulated data, as a function of the three coupling parameters k_12_, k_13_, and k_23_. In effect, we attempted to line up the locations of the poles and zeroes by optimising the three parameters. Once these coupling coefficients were estimated, they too were kept constant for all of the subsequent calculations. This ensured that the model could not over-fit to measurement data sets by changing parameters that are known to remain constant for a fixed coil array.

**Figure 10:**
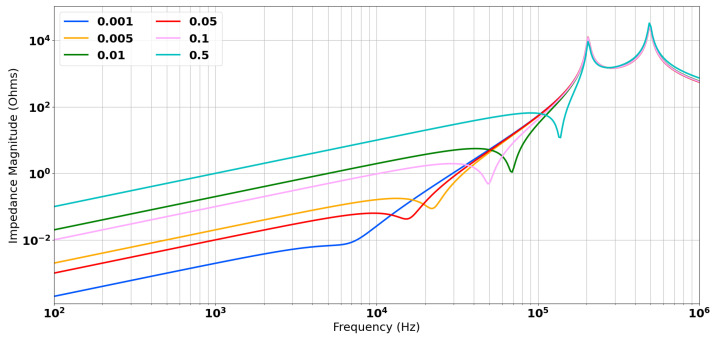
The impact of the tuning the value of ΔM on the location of the zero and the magnitude of the inductive coupling.

**Figure 11:**
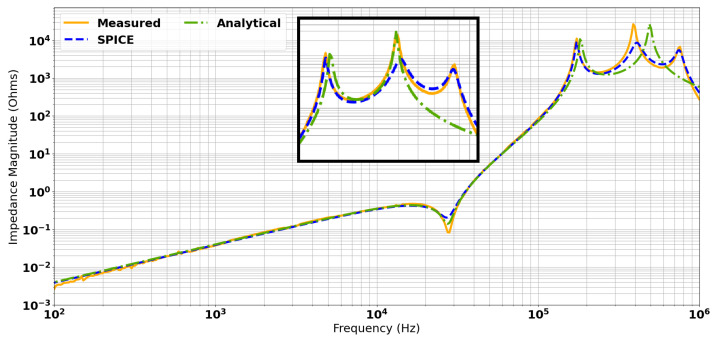
Comparison of measured, simulated, and calculated transimpedance spectra. Inset: The impact of adding capacitative loading added to the analytical model.

The analytical model has an additional parameter of ΔM. This was estimated by minimising the error between the measured location of the zero and the calculated value. Figure 10 shows how the zero term and the inductive coupling vary as a function of ΔM. The minimised value of ΔM was −0.02; this resulted in co-location of the zero term with the measured data and a good match of the inductive coupling. This is shown in Figure 11.

**Figure 12:**
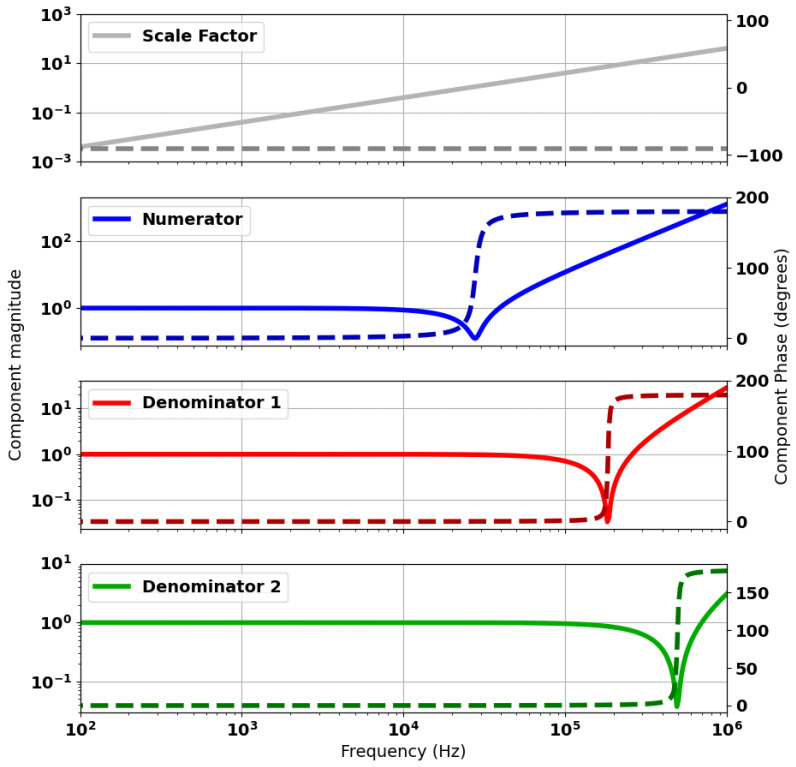
Spectral response of components of the analytical model as defined in (11). Component magnitudes are plotted as solid lines and respective phases as dashed lines.

Figure 12 shows the calculated spectral response for the numerator and denominator components of (11). In this case, the term “scale factor” refers to the jωΔM component of the model. “Numerator” refers to the evaluated numerator term of the equation. The responses for “Denominator 1” and “Denominator 2” relate to the terms $g_{2}\left(j\omega \right)$ and $g_{3}\left(j\omega \right)$, respectively. The response shows the location of the zero terms in each of these components. These correspond to zeroes in (11) for the “numerator” and poles for the “denominator” terms.

The estimated values of the coupling coefficients align with what would be expected. Rx_O_ and Tx are represented by k_13_. The coils are the same size and shape and are offset by 1 cm. It is, therefore, considered likely that the two coils are strongly coupled compared with the other coils in the network. The two coils are also wound in phase with one another, so this term is expected to be positive. Conversely, the Rx_I_ coil is wound in anti-phase with respect to Tx and Rx_O_. In which case, the negative values of k_12_ and k_23_ are as expected. It also aligns with the expectation that these coupling coefficients will have similar values to one another. This is because the similarity in geometries between the Tx and Rx_O_ coils and their similar locations with respect to Rx_I_ mean that the coupling should be approximately the same for each coil pair.

A comparison of the three data sets is shown in Figure 11 and the parameters extracted from these models are given in Table 1.

Figure 11 shows that there is good agreement with the estimation of coupling coefficients, given the coincident zero term and first pole for the “Measured”, “SPICE”, and “Analytical” data sets. The plots of the SPICE and measured data contain a third pole, which corresponds to the resonant frequency of the transmit coil. This is not considered in the analytical model as, for simplicity, parasitic capacitance within the transmit coil has been neglected. Compared with the other data sets, the second pole is approximately 100 kHz higher in the analytical model, although there is excellent agreement between all three data sets up to approximately 200 kHz. A possible explanation for this is that the outer receive coil and the transmit coil are strongly coupled to one another, as shown in Table 1. Therefore, it is very likely that there is a resonant interaction between these coils that is not accounted for by the analytical model.

The inset within Figure 11 shows that greater agreement could be reached by adding 145 pF across $L_{3}$ to align the resonant frequency with the measured values and account for the resonant loading. However, this correction was not applied to the analytical model, as it was considered to be over-fitting, and almost certainly incorrectly accounting for the full nature of the resonant interaction of the Tx and Rx_O_ coils. It is not expected that this system would be usable beyond the first resonant peak, and so complete agreement beyond ∼170 kHz, while desirable, is not practically necessary. The SPICE model shows excellent agreement with the measured data across the complete spectrum, and, therefore, provides a baseline scenario from which to simulate balancing methods.

**Figure 13:**
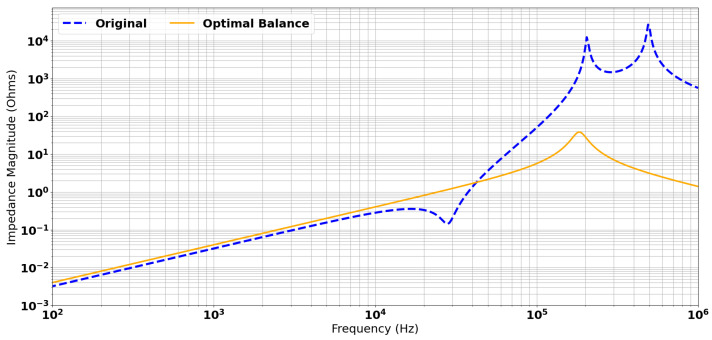
Ideal response of balanced resonances and Q-factors.

The analytical model was used to calculate the expected transimpedance spectrum under idealised conditions. This involved adding capacitance across $L_{3}$ to more closely match the resonant frequency and resistance across $L_{3}$ to align the Q-Factors. The response is shown in Figure 13. This plot verifies the cancellation of the pole–zero pair, and suggests that a system bandwidth of ∼100 kHz is achievable for the system. The model required the addition of 1490 pF and 2.62 kΩ in parallel with $L_{3}$ to achieve an ideal balance. Given the difference in resonant frequencies between the analytical model and the SPICE simulation, and the fact that the SPICE simulation contains a more realistic estimation for cable capacitance, it is not expected that these values can be directly applied to the real system. However, they do confirm the method and provide a target for the best achievable result using this process.

**Figure 14:**
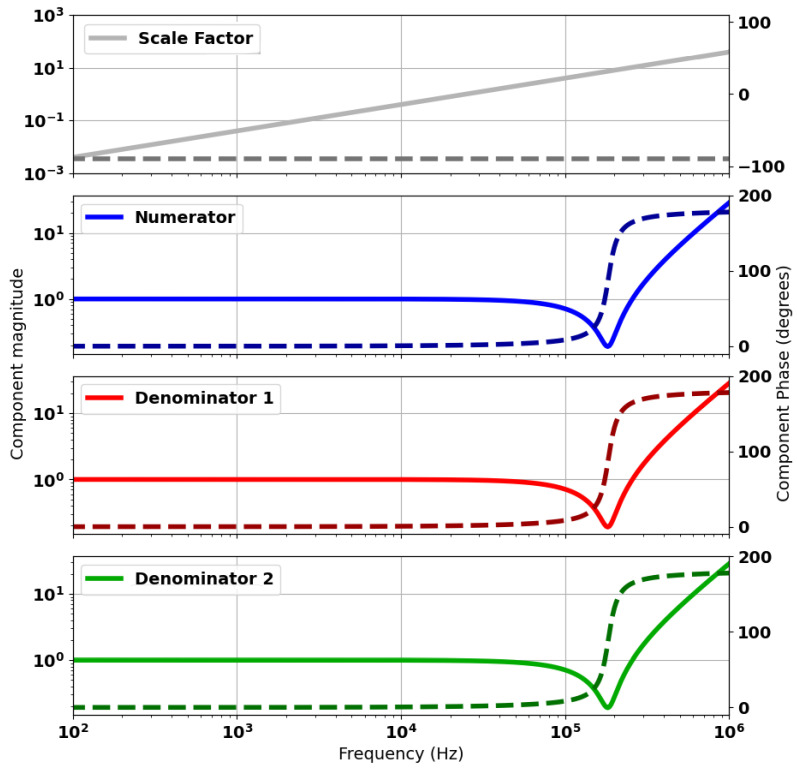
Spectral response of components of the analytical model for an ideally balanced head. Component magnitudes are plotted as solid lines and respective phases as dashed lines.

Figure 14 provides an explanation for this idealised balancing. The figure shows that by tuning the C and R parameters accordingly, the spectral response for the numerator and two denominator terms are identical. The residual error term is zero between the numerator and either of the denominator responses. Consequently, the pole–zero pair perfectly cancel, leaving the single pole from the remaining denominator term. This represents an idealised case where component tolerances and other error sources are not accounted for.

## 6. Experimental Validation of Coil Balancing Method

**Figure 15:**
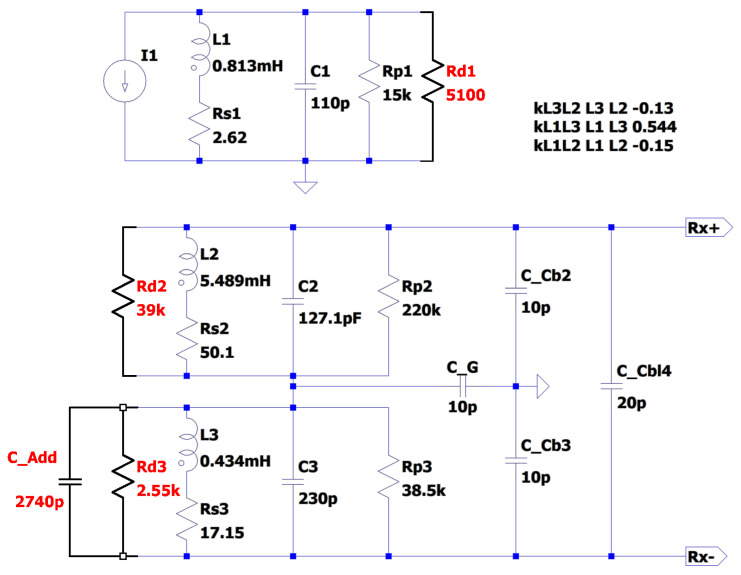
SPICE model of corrected transimpedance of the coil network.

The SPICE model was used as a means of testing possible balancing arrangements. This approach verified the ability to remove the pole–zero pair, albeit with slightly different capacitor and resistor values than estimated on account of the error in the model prediction of the second pole frequency. The simulation was able to reach the same baseline as the analytical model predicted in Figure 13, predicting a usable bandwidth of ∼100 kHz. Some fine tuning was needed in the experimental system due to the availability of component values and tolerances. The prediction of the SPICE model was taken as a starting point, and <100 pF of additional capacitance was required to achieve the best possible practical balance. This was determined empirically, where repeated transimpedance measurements were taken with small increments of capacitance added with each iteration. The optimal level of capacitance was determined as the amount that minimised the coupling between the coils. The component values used in the final balancing arrangement were updated in the SPICE model and are shown in Figure 15. The extra components that were added to the original, mis-balanced sensor system are shown in red. These additional components include Rdx terms, which are additional damping resistances, and a C_Add term, which represents the added capacitance required to align the poles.

**Figure 16:**
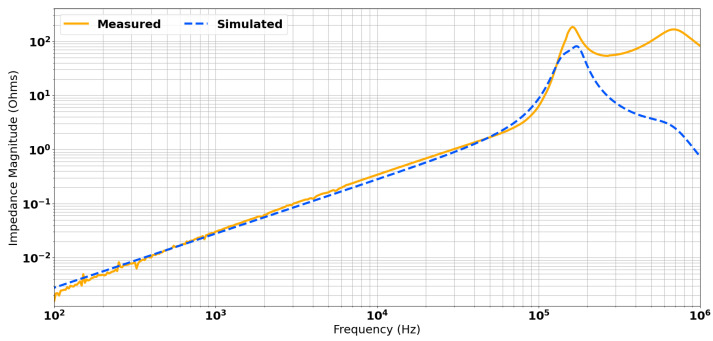
Comparison of measured and simulated transimpedance spectra post-balancing.

**Figure 17:**
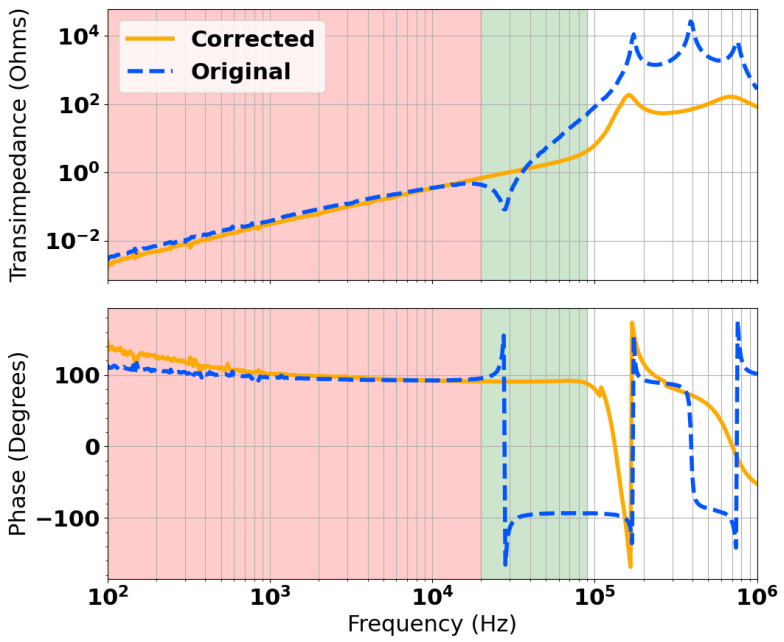
Comparison of the original and corrected impedance spectra showing an extended inductive region.

Figure 16 shows the measured transimpedance and the results of SPICE modelling. There is very good agreement between the two data sets up to around 200 kHz. There is some disagreement between the simulated and measured data sets, which shows more resonant interaction from the transmit coil than the model predicts. It is possible this could be accounted for by lumped equivalent components used to represent inductance, capacitance, and resistance, although in practice, these are spatially distributed. Nevertheless, as this is beyond the resonant frequency of the gradiometer, this does not impact the usable bandwidth of the sensor. Figure 17 shows a comparison of the measured transimpedance for the mis-balanced head and the result of the applied correction. The original bandwidth is highlighted in red and the new extended bandwidth is shown in green. It is possible to see that both magnitude and phase stability were achieved for approximately 90 kHz of bandwidth. This is sufficiently close to the predicted maximum of 100 kHz in Figure 13. It is also well beyond the desired maximum operating frequency of 50 kHz. It can also be noted that the resulting pole was also dampened by nearly two orders of magnitude, from the order of 10 kΩ to around 100 Ω.

## 7. Conclusions

The work presented in this paper demonstrates a viable method for extending the bandwidth of a mis-balanced differential coil pair. It achieves this through correcting the residual mis-balance in resonant terms and Q-factors of the receive coil network. The results show that a corrected system can operate across almost all of the spectrum up to a region just below that of the first system resonance, as is typically the case for a single damped inductor.

Although a specific coil geometry is presented here, the method is transferable to a wide range of sensor geometries and can be applied to other misbalanced systems in the same way. The corrections applied result in a number of positive changes to the measurement system, including extension of the system bandwidth by around 4.5×, a net reduction in background coupling at frequencies above the zero term and first resonant peak, and a damping of the resonances themselves.

The extension of the sensor bandwidth is a significant advantage for spectroscopic measurement systems. This is due to the fact that improving the range of frequencies a system can operate at allows for a greater understanding of a measured target. For instance, such systems can be used to characterise metallic targets, which are known to be frequency dependent [25].

It is anticipated that further magnetic balancing can be applied by breaking out a small number of turns from each coil, and spatially varying the coupling of these turns to further alter the transimpedance, although the correction applied in this paper is sufficient for use in the experimental system demonstrated.

The analytical model could be further developed to account for the resonant interaction of the transmit coil. While this does not limit this study, there is a clear divergence in agreement between the analytical model and the measured data set as frequencies approach the resonant frequency of the transmit coil. There is also the possibility to develop the complexity of the modelling, such that it goes beyond the first order lumped circuit model presented here. Such an approach would likely need to account for parasitic capacitance distributed over turns with multiple RLC terms. This would represent a significant escalation in model complexity from the model presented here. Expansion of the model to include this level of detail would undoubtedly improve the accuracy of the simulation, given it would provide a more realistic representation of the practical system. Although some empirical correction was needed for the system shown, this paper has shown that neglecting parasitic capacitances and using a first-order lumped circuit model can yield the desired results. Future work could likely remove the need for this empirical correction, and thereby reduce development timeed and improve theoretical understanding of the modelling of resonant differential coils.

## Figures and Tables

**Table 1:** Coupling coefficients extracted from the fitting process.

ΔM	=	−0.02	$k_{12}$	=	−0.150
$k_{13}$	=	0.544	$k_{23}$	=	−0.130

## Data Availability

The raw measurement data supporting the conclusions of this article will be made available by the authors upon request. This publication contains all information necessary to recreate the data simulated by the analytical model and/or SPICE simulation.

## Abbreviations

The following abbreviations are used in this manuscript:

NDT	Non-destructive testing
SNR	Signal-to-Noise ratio
SPICE	Simulation Program with Integrated Circuit Emphasis
